# Quality of life, pain and use of analgesic, anxiolytic and antidepressant medication, in people living in care homes

**DOI:** 10.1093/ageing/afae196

**Published:** 2024-09-06

**Authors:** Jemima T Collins, Lisa Irvine, Pip Logan, Katie Robinson, Erika Sims, Adam L Gordon

**Affiliations:** Centre for Rehabilitation and Ageing, Injury, Recovery and Inflammation Sciences Academic Unit, School of Medicine, the University of Nottingham, Nottingham, UK; University Hospitals Derby and Burton NHS Trust, Derby, UK; Centre for Research in Public Health and Community Care, University of Hertfordshire, Hatfield, UK; Centre for Rehabilitation and Ageing, Injury, Recovery and Inflammation Sciences Academic Unit, School of Medicine, the University of Nottingham, Nottingham, UK; Centre for Rehabilitation and Ageing, Injury, Recovery and Inflammation Sciences Academic Unit, School of Medicine, the University of Nottingham, Nottingham, UK; Research and Innovation, Nottingham University Hospitals NHS Trust, Nottingham, UK; Norwich Medical School, University of East Anglia, Norwich, UK; Centre for Rehabilitation and Ageing, Injury, Recovery and Inflammation Sciences Academic Unit, School of Medicine, the University of Nottingham, Nottingham, UK; University Hospitals Derby and Burton NHS Trust, Derby, UK; Applied Research Collaboration-East Midlands (ARC-EM), Nottingham, UK

**Keywords:** pain, analgesia, care homes, quality of life, older people

## Abstract

**Background:**

People living in care homes often have problems with pain, anxiety and depression. Whether being on analgesia, anxiolytics or antidepressants has any bearing on pain severity and quality of life (QoL) in this population, requires further investigation.

**Objectives:**

(i) to examine the relationship between pain, anxiety and depression and medication use in care home residents and (ii) to compare those on medications to treat pain, anxiety and depression, and those who were not, and associations with pain severity and overall QoL.

**Methods:**

This was a secondary analysis of a randomised controlled trial testing a falls prevention intervention in care homes. We recorded pain, anxiety and depression, QoL measurements and prescribed medication use.

**Results:**

In 1589 participants, the mean age was 84.7 years (±9.3 SD), 32.2% were male and 67.3% had a diagnosis of dementia. 54.3% and 53.2% of participants had some level of pain and anxiety or depression respectively, regardless of prescribed medication use. There was a direct association between pain severity and being on any analgesia, opioid analgesia, and antidepressants, but no associations between pain severity and use of paracetamol and anxiolytics. QoL was best for residents with no pain and not on any analgesia, anxiolytics or antidepressants and worst for those with moderate-extreme pain and taking at least two of these classes of medications.

**Conclusion:**

Many care home residents live with pain, anxiety and depression. Addressing residents’ pain may also increase their quality of life, but using medication alone to reach this goal may be inadequate.

## Key Points

- Over 50% of people living in UK care homes have pain.
- There is a significant association between the severities of pain and anxiety and depression in people living in care homes
- Higher pain severity was associated with taking any analgesia, opioid analgesia and antidepressants.
- Quality of life was lowest for care home residents with greatest pain and was on ≥2 analgesia, antidepressants or anxiolytics.

## Introduction

Older people living in care homes have high care needs due to cognitive and physical disabilities [1, 2]. There are ~11 000 care homes in the UK providing full time care for >400 000 residents who have dependency, and whose care needs cannot be met at home [3].

Pain is sub-optimally assessed and treated, in ageing populations [4, 5]. An estimated 40%–50% of older people living in care homes have troublesome pain [6] and this proportion can be as high as 79% in those living with dementia and at the end of life [7, 8]. Pain is closely associated to, and frequently coexists with, neuropsychiatric symptoms such as anxiety, depression and behavioural symptoms. A number of studies have considered whether it is beneficial to use analgesics to treat common neuropsychiatric symptoms, anxiety and depression and of using anxiolytics and antidepressants to treat pain. However, evidence of efficacy has been mixed [9–12].

Health-related quality of life (QoL) is one of the most important measurable parameters in the evaluation of pain management success, and validated scales for care home residents include questions regarding pain, feelings and physical functioning [13, 14]. Neuropsychiatric symptoms may be caused by underlying pain, directly affect both pain and QoL, or mediate the relationship between pain and QoL [15–17]. This study set out to examine the relationship between pain, anxiety and depression in this population. Moreover, we sought to evaluate whether there was any difference between the pain levels and overall QoL of care home residents who were on medications to treat pain, anxiety and depression, compared with those who were not.

## Methods

### Study design, setting, sample size and intervention

We performed a cross-sectional secondary analysis of data collected from the Falls in Care Homes (FinCH) trial [18]. FinCH was a multicentre, parallel, 1:1 cluster randomised controlled trial (RCT) to test the Guide to Action Care Home (GtACH) programme (a risk assessment and decision support tool which provided falls prevention training and resources to all care home staff) compared with standard care. The FinCH RCT was given ethical approval by the Yorkshire and Humber–Bradford Leeds research ethics committee (REC 16/YH/0111) and recruited 1657 participants from 84 care homes between November 2016 and January 2018 [19]. Its main aim was to determine both clinical and cost effectiveness of GtACH compared with usual care [18].

### Outcome measurements

i) Quality of Life

DEMQOL-P is a 31-item questionnaire completed by the primary caregiver as a proxy for a person living with dementia [14]. Scores range from 31 to 124, with higher scores reflecting better health-related QoL. DEMQOL-P was developed specifically for people living with dementia but is widely used and accepted to measure QoL in all care home residents [14, 20]. For the purposes of this study, QoL was defined as baseline DEMQOL-P scores.

ii) Pain severity

Pain measurements were extracted from the corresponding domains in the EuroQoL-5 domain-5 level-Proxy (EQ-5D-5L-P) [21] questionnaires completed as part of the study. There are 5 levels for pain: 1 = No pain or discomfort; 2 = Slight pain or discomfort; 3, 4 and 5 = Moderate, Severe and Extreme pain or discomfort, respectively. Because we analysed subdomains of the EQ-5D-5L-P for pain and anxiety we did not use the overall EQ-5D-5L-P score as a measure of QoL in this secondary analysis.

### Exposure

The exposure in this study is defined as types of prescribed medications. Medication use was noted from care home medication records. For this secondary analysis, we specifically focused on medications classified in Chapter 4 of the British National Formulary [22] as analgesia, anxiolytics and antidepressants (collectively referred to as ‘medication’ hereafter).

### Covariates

The covariates in this study are defined as residents’ age, sex, dementia diagnosis, Barthel index and severity of anxiety or depression as measured in the EQ-5D-5L-P [21]. There are five levels for anxiety and depression: 1 = Not anxious or depressed; 2 = Slightly anxious or depressed; 3, 4 and 5 = Moderately, Severely or Extremely anxious or depressed, respectively.

### Data extraction and statistical analysis

All data extracted from the main FinCH dataset for the purposes of this secondary analysis were baseline measurements. The rationale for this was to enable cross-sectional analysis of pain, depression and anxiety levels of care home residents, correlations with medications and QoL, and to reduce longitudinal data missingness due to attrition.

Descriptive statistics were expressed as absolute and relative frequencies for discrete variables, and means and standard deviation for continuous variables. The distribution of each variable was assessed for normality with the Kolmogorov–Smirnov and the Shapiro–Wilks tests. We used the Pearson correlation test to evaluate for between group differences of prevalence for categorical data, and Kruskal Wallis test for differences between groups of non-parametric continuous data. To visually assess the difference for primary outcomes (QoL and pain severities), a scatterplot was used. IBM SPSS statistics (version 28) was used for statistical analysis.

## Results

Out of 1657 participants from the original FinCH dataset, 1589 residents (95.9%) with complete baseline pain data were included into this study. The average (SD) participant age was 84.7 years (±9.3), 32.2% were male and 67.3% had a diagnosis of dementia. The average (SD) Barthel Index was 8.6 (6.0) (Table 1).

**Table 1:** Characteristics and descriptive statistics of care home residents with complete pain data included in the study

	All Residents (n = 1589)	No Pain (n = 726)	Mild Pain n = 508)	Moderate Pain (n = 278)	Severe Pain (n = 60)	Extreme Pain (n = 17)	Pearson’s correlation coefficient (r), p-value
Age in years, Mean ± SD^§^	84.7 ± 9.3	83.8 (9.4)	85.3 (8.8)	85.6 (9.8)	86.6 (7.5)	84.7 (7.5)	r 0.081, p = 0.001
Sex Males, n (%) Females, n (%)	512 (32.2) 1077 (67.8)	253 (34.8) 473 (65.2)	161 (31.7) 347 (68.3)	76 (27.3) 202 (72.7)	42 (70) 18 (30)	13 (76.5) 4 (23.5)	r − 0.058 p = 0.021
Barthel Index Mean ± SD^§^	8.6 ± 6.0	9.4 (6.1)	8.1 (5.8)	7.8 (6)	6.3 (5.2)	6.2 (6.3)	r − 0.14 p < 0.001
Dementia diagnosis, n (%) No Yes Missing	518 (32.6) 1070 (67.3) 1 (0.1)	202 (27.8) 524 (72.2)	169 (33.3) 338 (66.7)	111 (39.9) 167 (60.1)	33 (55) 27 (45)	3 (17.6) 14 (82.4)	r − 0.108 p < 0.001
Presence of anxiety/depression, EQ5D5L, n (%) Not anxious/depressed Slightly anxious/depressed Moderately anxious/depressed Severely anxious/depressed Extremely anxious/depressed Missing	744 (46.8) 502 (31.6) 235 (14.8) 68 (4.3) 38 (2.4) 2 (0.1)	465 (64.2) 176 (24.3) 55 (7.6) 19 (2.6) 9 (1.2)	180 (35.4) 206 (40.6) 89 (17.5) 21 (4.1) 12 (2.4)	81 (29.1) 93 (33.5) 75 (27) 19 (6.8) 10 (3.6)	12 (20) 24 (40) 13 (21.7) 7 (11.7) 4 (6.7)	6 (35.3) 3 (17.6) 3 (17.6) 2 (11.8) 3 (17.6)	r 0.309 p < 0.001
Number of prescribed analgesic medications, n (%) 0 1 or more	768 (48.3) 821 (51.7)	396 (54.5) 330 (45.5)	235 (46.3) 273 (53.7)	120 (43.2) 158 (56.8)	14 (23.3) 46 (76.7)	3 (17.6) 14 (82.4)	r 0.144 p < 0.001
Paracetamol use, n (%) No Yes	1002 (63.1) 587 (36.9)	470 (64.7) 256 (35.3)	312 (61.4) 196 (38.6)	182 (65.5) 96 (34.5)	29 (48.3) 31 (51.7)	9 (52.9) 8 (47.1)	r 0.04 p = 0.110
Opioid Medication use, n (%) 0 1 or more	1183 (74.4) 406 (25.6)	598 (82.4) 128 (17.6)	377 (74.2) 131 (25.8)	179 (64.4) 99 (35.6)	22 (36.7) 38 (63.3)	7 (41.2) 10 (58.8)	r 0.231 p < 0.001
Anxiolytic Medication use, n (%) 0 1 or more	1408 (88.6) 181 (11.4)	652 (89.8) 74 (10.2)	442 (87) 66 (13)	246 (88.5) 32 (11.5)	53 (88.3) 7 (11.7)	15 (88.2) 2 (11.8)	r 0.021 p = 0.413
Antidepressant Medication use, n (%) 0 1 or more	878 (55.3) 711 (44.7)	422 (58.1) 304 (41.9)	285 (56.1) 223 (43.9)	140 (50.4) 138 (59.6)	25 (41.7) 35 (58.3)	6 (35.3) 11 (64.7)	r 0.084 p < 0.001

A total of 54.3% had pain and 53.2% had anxiety or depression. There was a significant correlation between severity of pain, and severity of anxiety or depression (Pearson’s r = 0.309). In those not taking regular analgesia, 48% had some level of pain. In those taking one or more analgesic, 59.9% had some level of pain. There was an association between pain severity and being prescribed any analgesic, opioids and antidepressants, but no associations between pain severity and use of paracetamol or anxiolytics.

**Figure 1:**
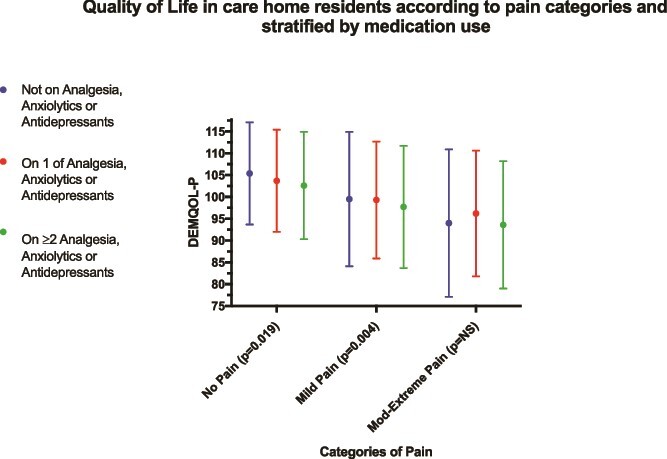
Quality of life (DEMQOL-P score) of care home residents, comparing differences between groups not prescribed any analgesia, anxiolytics or antidepressants, only prescribed one class of analgesia, anxiolytics or antidepressants, or prescribed both analgesia and anxiolytic or antidepressant; stratified according to pain severity. Kruskal Wallis test used to compare between groups differences.

The mean (SD) DEMQOL-*P* values in the different pain severity categories were 103.9 (11.9), 98.7 (14.2) and 94.6 (15) for groups of no pain, mild pain and moderate to extreme pain, respectively. Within the no pain and mild pain categories, people who were on two or more classes of medication had significantly worse QoL compared with those on 0 or 1 class only, but in the moderate to extreme category there was no significant difference in QoL stratified by medication use (Figure 1). Supplementary Tables 1 and 2 contain regression analyses in which pain is found to be significantly inversely associated with QoL, even after adjusting for confounding factors.

## Discussion

Pain is a common problem in ageing populations. This secondary analysis of a large randomised controlled trial dataset of care home residents adds insight into medication use in this population and associations with pain. Higher pain severities were associated with higher medication use, in keeping with previous literature [23]. Although there is an association between increased use of opioids and increased pain, this does not necessarily mean that opioids are not effective in residents with higher levels of pain. It may be that their pain was much higher to begin with, and the use of opioids has mitigated this severity. Paracetamol is often used in combination with other stronger analgesics and likely contributes to overall analgesia effectiveness.

There is modest evidence that taking antidepressant, anxiolytic and analgesic medications may improve QoL [24–26] but this is less clear in care home residents [10]. In this study, residents on two or more medications from analgesia, antidepressant and anxiolytic categories with moderate to extreme pain had the lowest QoL. This data suggests that pain is important in influencing QoL, regardless of medication use. This emphasizes that initiating and continuing these medications should be based on clinical indication, ongoing need, and the risk and benefit profile for the individual.

Over 50% of care home residents had some level of pain, in keeping with other literature suggesting that under-treatment of pain in care home residents is due to under-recognition of pain [27]. Assessment of medication efficacy could be improved by care home staff intentionally assessing pain with validated assessment tools [28]. Clinicians may then review medications, and others involved in the residents’ care such as the Activity Coordinator may suggest non-pharmacological approaches such as tailored pain interventions and music therapy [29].

### Strengths and limitations

This study effectively repurposed individual participant data from a large RCT conducted in UK care homes to compare QoL measures and medication use, providing insights not typically available through administrative data [30]. These care homes were ‘research ready’ and thus might be more proactive than other facilities in performing medication reviews. All data were taken at baseline and as such there was very little missing data. Due to the cross-sectional, observational nature of this study, the cause and effect relationship could not be established and thus translation to causality remains speculative. A further limitation was that we did not note doses, frequencies or as required medication use as this was not fully recorded. ‘Medication use’ referred to prescribed analgesia, anxiolytic or antidepressant medication only; full compliance was assumed but not confirmed. Noting the reasons for pain may have given clarity to, for example, the effectiveness of medication taken for neuropathic pain and/or depression. Access to these data would have strengthened the accuracy of our findings.

## Conclusion

This study highlights that a substantial proportion of care home residents live with pain. Nearly half of those not prescribed any analgesia had pain, representing a subset of residents who almost certainly would benefit from either non-pharmacological and/or pharmacological interventions. Addressing residents’ pain may also increase quality of life, but using medication alone to reach this goal is likely to be inadequate.

## Supplementary Material

aa-24-0838-File002_afae196
