# Supplementary material for: Quality of life, pain and use of analgesic, anxiolytic and antidepressant medication, in people living in care homes
Source: Age Ageing. 2024 Sep 6;53(9):afae196. doi: 10.1093/ageing/afae196 (PMC11377180; doi:10.1093/ageing/afae196)
Supplement: aa-24-0838-File002_afae196 [file aa-24-0838-file002_afae196.docx]

Supplementary Tables for Age and Ageing publication.

Title: Quality of Life, Pain and Use of Analgesic, Anxiolytic and Antidepressant medication, in people living in care homes.

| **Variables** | **Unstandardised Beta** | **95% Confidence Intervals** |
| --- | --- | --- |
| **Model 1** | | |
| Pain severity | -3.947** | -4.665 to -3.228 |
| Age | -0.067 | -0.139 to 0.006 |
| Gender | 0.460 | -0.976 to 1.896 |
| **Model 2** | | |
| Pain severity | -4.165** | -4.904 to -3.426 |
| Age | -0.099* | -0.172 to -0.025 |
| Gender | 0.426 | -1.011 to 1.863 |
| 0, 1 or 2 or more Analgesia medications | 0.509 | -0.461 to 1.479 |
| 0, 1 or 2 or more Anxiolytic medications | -3.512** | -5.459 to -1.565 |
| 0, 1 or 2 or more Antidepressant medications | -2.228** | -3.327 to -1.129 |
| Dementia diagnosis | -2.313* | -3.741 to -0.886 |
| Barthel index | -0.111 | -0.223 to 0.000 |

*Supplementary Table 1: Linear Regression Models of Pain severity and continuous DEMQOL-P scores, adjusted for age, gender, number of analgesia, anxiolytic, and antidepressant medications, dementia, and Barthel index. *p<0.01 **p<0.001*

| **Variables** |  | **Beta** | **95% Confidence Intervals** |
| --- | --- | --- | --- |
| **Model 1** | | | |
| Age |  | 0.001 | 0.990 to 1.013 |
| Gender |  | -0.063 | 0.753 to 1.171 |
| Pain | No Pain |  |  |
|  | Slight Pain | -0.509** | 0.475 to 0.759 |
|  | Moderate Pain | -0.707** | 0.372 to 0.654 |
|  | Severe Pain | -0.974** | 0.221 to 0.644 |
|  | Extreme Pain | -1.314* | 0.098 to 0.736 |
| **Model 2** | | | |
| Age |  | 0.001 | 0.989 to 1.013 |
| Gender |  | -0.114 | 0.708 to 1.125 |
| Pain | No Pain |  |  |
|  | Slight Pain | -0.432** | 0.507 to 0.831 |
|  | Moderate Pain | -0.637** | 0.392 to 0.713 |
|  | Severe Pain | -0.877* | 0.235 to 0.735 |
|  | Extreme Pain | -1.096* | 0.116 to 0.961 |
| Analgesia | Not on analgesia |  |  |
|  | Taking 1 analgesia medication | 0.021 | 0.812 to 1.285 |
|  | Taking 2 or more analgesia medications | 0.177 | 0.844 to 1.687 |
| Anxiolytics | Not on anxiolytics |  |  |
|  | Taking 1 analgesia medication | -0.269 | 0.543 to 1.075 |
|  | Taking 2 or more analgesia medications | 0.152 | 0.271 to 5.00 |
| Antidepressants | Not on antidepressants |  |  |
|  | Taking 1 antidepressant medication | -0.108 | 0.716 to 1.125 |
|  | Taking 2 or more antidepressant medications | 0.065 | 0.673 to 1.692 |
| Dementia diagnosis | Yes or no | -0.292* | 0.59 to 0944 |
| Barthel index |  | 0.089** | 1.073 to 1.114 |

*Supplementary Table 2: Binary Logistic Regression Models of Pain severity and the Summary DEMQOL-P question “How would you say [the resident] would rate his/her quality of life overall?”. Fair and Poor vs Good and Very good outcomes adjusted for age, gender, number of analgesia, anxiolytic, and antidepressant medications, dementia, and Barthel index. *p<0.05 **p<0.001*
